# TLFS23 Tamil language fingerspelling dataset

**DOI:** 10.1016/j.dib.2023.109961

**Published:** 2023-12-15

**Authors:** Bavesh Ram S, Chirranjeavi M, Aaruran S, Gokulraj VA, Binoy B Nair, Harikumar M．E

**Affiliations:** Department of Electronics and Communication Engineering, Amrita School of Engineering, Amrita Vishwa Vidyapeetham, Coimbatore, India

**Keywords:** Indian sign language, Tamil, Computer vision, Image dataset

## Abstract

Tamil is one of the oldest existing languages, spoken by around 65 million people across India, Sri Lanka and South-East Asia. Countries such as Fiji and South Africa also have a significant population with Tamil ancestry. Tamil is a complex language and has 247 characters. A labelled dataset for Tamil Fingerspelling named TLFS23 has been created for research related to vision-based Fingerspelling translators for the Speech and hearing Impaired. The dataset would open up avenues to develop automated systems as translators and interpreters for effective communication between fingerspelling language users and non- users, using computer vision and deep learning algorithms. One thousand images representing each unique finger flexion motion for every Tamil character was collected overall constituting a large dataset with 248 classes with a total of 2,55,155 images. The images were contributed by 120 individuals from different age groups. The dataset is made publicly available at: https://data.mendeley.com/datasets/39kzs5pxmk/2.

Specifications Table

**Table:** 

Subject	Computer Science
Specific subject area	Tamil Fingerspelling, Deep Learning, Computer Vision
Data format	Raw
Type of data	Image (640×480 pixels JPG format)
Data collection	A fixed focus, 0.9 Megapixel webcam (Logitech C270) was used to capture the images. A python-based program using OpenCV library was used to acquire the images and store them into one of the 248 folders as required. The dataset was collected during the period February 2023–June 2023 from 120 volunteers.
Data source location	Amrita School of Engineering, Coimbatore, Amrita Vishwa Vidyapeetham, India
Data accessibility	Repository name: Mendeley Data Data identification number: 10.17632/39kzs5pxmk.2 Direct URL to data: https://data.mendeley.com/datasets/39kzs5pxmk/2 Instructions for accessing these data: The data publicly available at Mendeley data. The dataset can be directly downloaded and the images will be available. The files associated with this dataset are licensed under a Creative Commons Attribution 4.0 International licence.

## 1 Value of the Data

• Vision based fingerspelling and sign language translators would play a huge role in bridging the gap in communication for the speech impaired. As Tamil language is spoken by over 65 million people worldwide [1], a Tamil fingerspelling dataset has great significance. The TLFS23 is a large Tamil fingerspelling dataset representing 247 Tamil characters with 1000 image samples for each character (an additional 1000 images are taken only for the background, without any hand in the frame), resulting in a total of 2,55,155  images. The link to the dataset is available in Ref. [2].
• This dataset will enable effective training of deep learning and computer vision models for Tamil fingerspelling recognition. The dataset can be used Healthcare, Education Technology, Assistive technology and many more effectively.
• The Dataset comprises data collected from volunteers belonging to a wide age group, from 15 years old to 80 years old individuals presenting data with a great variation which includes variation in finger length, finger skin tone, and finger flexibility.
• The TLFS23 is fully-labelled so that it can be used in vision-based automation of fingerspelling recognition for the speech and hearing impaired. An example is the use case of employing sign language recognition system for communicating with speech impaired patients during video- based consultations with doctor in telemedicine application as illustrated in Ref. [3].

## 2 Data Description

Though the availability of image datasets for Indian sign language [4,5] and American sign language [6,7] is abundant, the availability and knowledge of sign language and fingerspelling in vernacular languages is scarce. Studies such as [8] have demonstrated that fingerspelling training can have a significant positive impact on the learning abilities of deaf children by helping strengthen their word decoding and recognition skills. Fingerspelling has also been observed to assist the learning process for deaf students enrolled in further education colleges, as reported in Ref. [9]. However, the lack of fingerspelling datasets for vernacular languages such as Tamil is an impediment to development of recognition systems that can help common people interact more effectively with the speech impaired. Tamil is a prominent language in the Indian subcontinent and is considered to be one of the oldest languages. We have created a Tamil language fingerspelling image dataset to encourage users to develop Deep learning and computer vision models and translators to aid the speech and hearing impaired communicate in Tamil.

One of the major reasons for creating this image-based dataset is the fact that vision based systems are easier to deploy since the signer does not have to wear any specialized equipment such as a glove, as in Ref. [6].

We have created a large original image dataset that includes all the 247 characters of Tamil language which is broadly categorized into three divisions namely Uyir ezuthukal (vowels), Mei ezuthukal (consonants) and Uyirmei ezuthukal (combination of an Uyir ezuthu and a Mei ezuthu).

The TLFS23 dataset has been categorized into Uyir Ezuthukal, Mei Ezuthukal and Uyir Mei Ezuthukal. The Uyir Mei ezuthukal are the combination of the Uyir ezuthukal and Mei ezuthukal. The language consists of 12 Uyir ezuthukal that are considered vowels and 18 Mei ezuthukal considered consonants. Tamil Language presents us with a unique set of 216 Uyir Mei ezuthukal characters that are the combination of the Uyir and Mei ezuthukal characters. One special character called the Ayudha ezuthu is also available resulting in a total of 247 (12 Uyir ezuthukal +18 Mei ezuthukal +1 Ayudha ezuthu + (12 Uyir ezuthukal *18 Mei ezuthukal) characters. Table 1 shows all the 247 characters and their finger spelled signs and one background considered as the 248 categories, and their folder names as per the dataset repository. The data repository has 248 folders with each folder having over 1000 images falling under that category. The folders have a simple arrangement, no additional subfolders are present, except the folders mentioned in the Table 1.

**Table 1:** Table represents the characters, finger spelled signs and their folder name in the data repository.

**Fig. 1:**
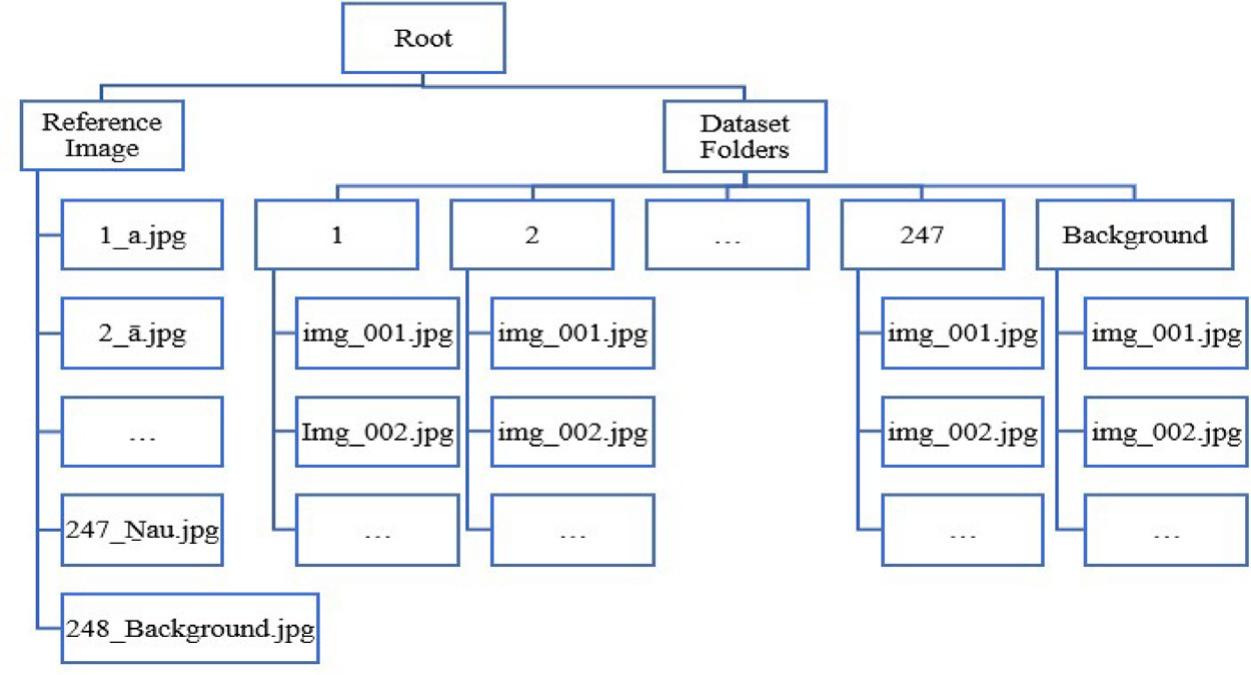
Folder layout for the TLFS23 Tamil language fingerspelling dataset.

The folder layout used for the TLFS23 Tamil language fingerspelling dataset is summarized in Fig. 1.

## 3 Experimental Design, Materials and Methods

### 3.1 Experimental Setup

**Fig. 2:**
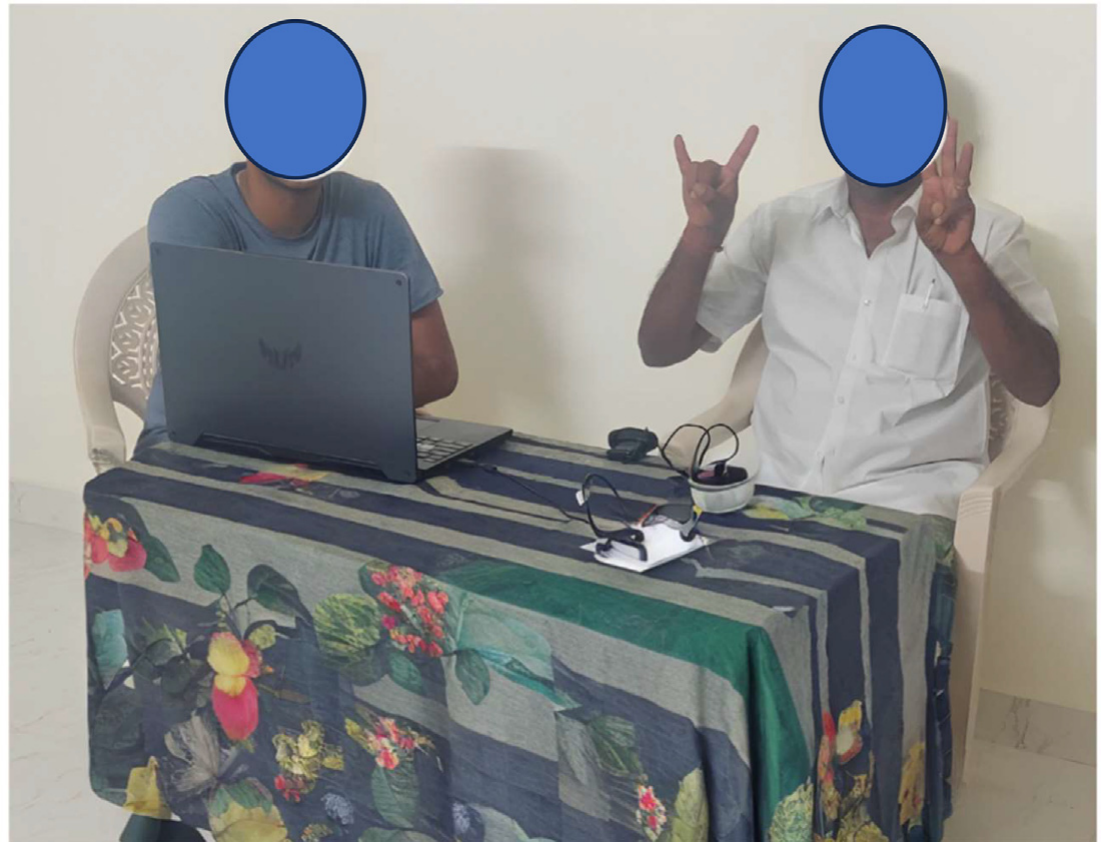
Setup facilitated for collecting images.

A setup for collecting the dataset as shown in the image was facilitated as shown in Fig. 2. The background for each image was mostly preferred to be a plain light background. The participants were drawn from the general population with the age and sex distribution as shown in Table 2. The participants were seated in a chair and the camera placed in front of them collected images. A laptop screen with the reference image for each character was always on display as it would be difficult for the participants to remember all the 247 characters. 2480 images were collected from each participant.

**Table 2:** Total number of participants and their diversity.

Age group	Male	Female	Total
10–30	21	23	44
31–45	24	18	22
46–60	11	11	20
> 60	7	5	12
**Total**	**63**	**57**	**120**

**Fig. 3:**
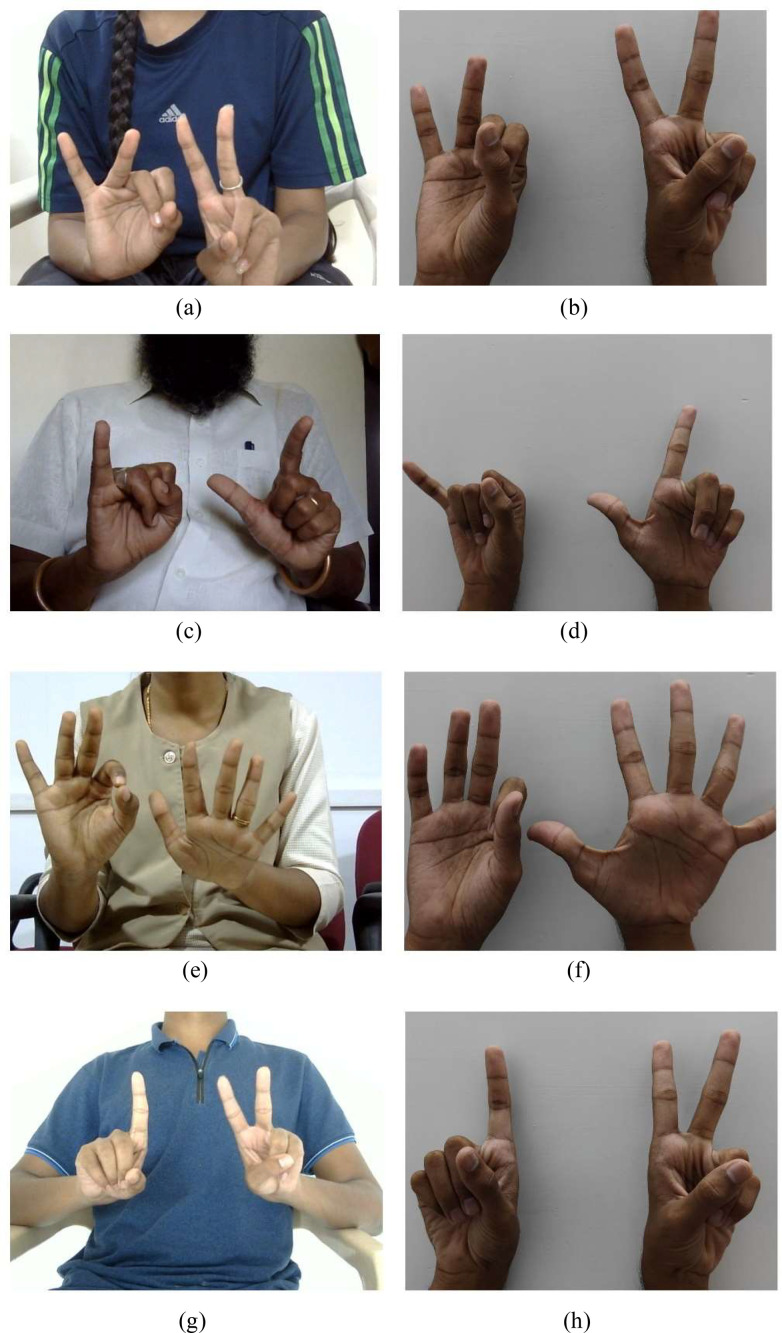
Data collection examples: images logged in the dataset represented in (a), (c), (e) and (g). Corresponding reference images are shown in (b), (d), (f) and (h), respectively. Fig. 3 (a), (b) represent the Tamil character Ñi; (c) and (d) represent the character Ngā; (e) and (f) represent Sē; (g) and (h) represent Ni.

The reference images and the corresponding images recorded for four volunteers (two male and two female) is shown in Fig. 3. The images logged in the dataset are represented in (a), (c), (e) and (g) while the corresponding reference images are shown in (b), (d), (f) and (h), respectively. Fig. 3 (a), (b) represent the Tamil character *Ñi*; (c) and (d) represent the character *Ngā*; (e) and (f) represent *Sē*; (g) and (h) represent *Ni*.

### 3.2 Dataset Generation Methodology

The data generation procedure can be summarized using the algorithm below:

Start

Step 1: Display reference image

Step 2: Volunteer demonstrates fingerspelling character

Step 3: Supervisor clicks button to accept image

Step 4: Log image as part of dataset

Step 5: If number of accepted images = 10

Step 6: Automatically change reference image to next finger-spelling character.

Step 7: Go to step 2

End

**Fig. 4:**
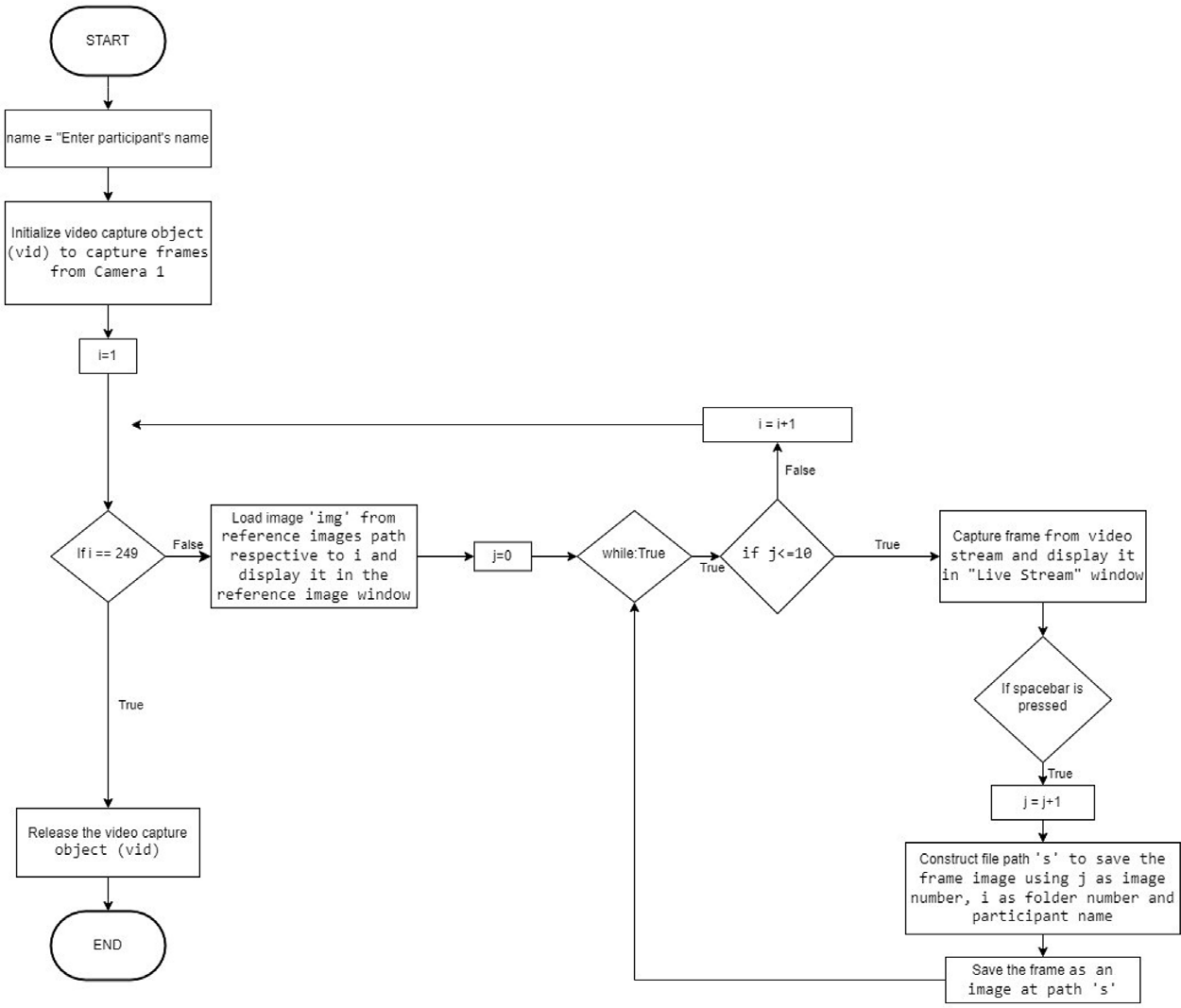
Flowchart of the code used for data acquisition.

The images were captured through a fixed focus, 0.9 Megapixel webcam (Logitech C270) controlled through a Python program which ensured that the collected data was automatically categorized into the 248 folders (247 folders characters and one folder for background). The flowchart of the code used for acquiring the image dataset is given in Fig. 4.

Fingerspelling images collected for each Tamil character has been stored in individual folders, for example, all the fingerspelling images for the Tamil alphabet character ‘ā’ have been put in the folder labelled ‘2’. The Tamil character and the corresponding folder names for all the Tamil alphabet characters has been listed in Table 1 for easy reference. The folder layout used in the data repository has also been illustrated in Fig. 1.

A total of 120 volunteers participated in the data compilation. The participants were diverse in terms of age and gender as shown in Table 2. The selection of the number of volunteers and their age distribution was derived from the information provided in the last official census conducted by Government of India in 2011.

The collected data from the participants had some images that were unsuitable which we termed as trash images. These trash images were sometimes formed due to a blur in the camera or wrong poses, or any humane error by the photographer or the participant. These images were filtered to remove these trash images and do not form the part of this dataset. These trash images were replaced by images from another 20 participants. Though the images collected were not equally distributed in all categories, they aided in the compiling a minimum of 1000 images in each category.  All images are scaled down to 640×480 pixels.

## Limitations

None.

## Ethics Statement

Informed consents have been obtained from all participants who aided us in the image collection. No human or animal studies were carried out. No social media data has been collected for the dataset. It is also worth noting that the images collected do not contain any sensitive information about the subject's identity.

## CRediT authorship contribution statement

**Bavesh Ram S:** Conceptualization, Methodology, Software, Validation, Investigation, Writing – original draft, Writing – review & editing, Project administration, Data curation, Formal analysis. **Chirranjeavi M:** Conceptualization, Software, Validation, Data curation, Writing – review & editing, Visualization, Formal analysis. **Aaruran S:** Formal analysis. **Gokulraj VA:** Formal analysis. **Binoy B Nair:** Supervision, Writing – review & editing. **Harikumar M．E:** Supervision, Writing – review & editing.

## Data Availability

TLFS23 - Tamil Language Finger Spelling Image Dataset (Original data) (Mendeley Data).

## References

[1] Annamalai E., Asher R.E. (2015).

[2] Chirranjeavi M., Bavesh Ram S., Varatharajan G., Sundaresh A, Nair B.B., Harikumar M.E (2023). TLFS23 - Tamil language finger spelling image dataset. Mendeley Data.

[3] Wazalwar S., Shrawankar U. (2020). Online healthcare consultation system for deaf & dumb during pandemic situation. Biosci. Biotechnol. Res. Commun..

[4] Sridhar A., Ganesan R.G., Kumar P., Khapra M. (2020). Proceedings of the 28th ACM International Conference on Multimedia, Association for Computing Machinery.

[5] Teja Mangamuri L.S., Jain L., Sharmay A. (2019). Proceedings of the IEEE International Conference on Issues Challenges in Intelligent Computing Techniques ICICT 2019.

[6] Duarte A., Palaskar S., Ventura L., Ghadiyaram D., DeHaan K., Metze F., Torres J., Giro-I-Nieto X. (2021). Proceedings of the IEEE Computer Society Conference on Computer Vision and Pattern Recognition.

[7] Vaezi Joze H., Koller O. (2019). Proceedings of the British Machine Vision Association.

[8] Ormel E., Giezen M.R., Knoors H., Verhoeven L., Gutierrez-Sigut E. (2022). Predictors of word and text reading fluency of deaf children in bilingual deaf education programmes. Languages.

[9] Iturriaga C., Young A (2022). Deaf students’ translanguaging practices in a further education college: Situating the semiotic repertoire in social interactions. J. Deaf Stud. Deaf Educ..

